# Consumer impulse buying behavior: the role of confidence as moderating effect

**DOI:** 10.1016/j.heliyon.2022.e09672

**Published:** 2022-06-08

**Authors:** Van Dat Tran

**Affiliations:** Faculty of Business Administration, Banking University, Ho Chi Minh, Viet Nam

**Keywords:** Social comparison, Materialism, Negative affect, Impulse buying, Confidence

## Abstract

Many indicators have been proposed that can contribute to impulse buying. However, few studies have examined the role of social comparison in impulse buying, materialism, and negative affect, and even less is known about the underlying processes that may moderate these relationships. The objective of this study was to create a framework that included social comparison, materialism, negative affect, impulse buying, and the moderator variable confidence in Vietnamese e-commerce. A total of 249 completed questionnaires were received from young people who frequently shop online. The study used a structural model and experimentally analyzed the links between materialism, social comparison, impulse buying, and negative affect, and how the moderating variable confidence influenced these interactions. The study finds that social comparison has a significant influence on materialism but has no impact on negative affect. However, negative affect significantly influences impulse buying. Materialism also has an impact on negative affect and impulse buying. Additionally, confidence has a beneficial moderating effect on the relationship between social comparison and impulse buying as well as social comparison and materialism. The limitations and implications of both the scientific and managerial aspects of the study were also addressed. The results will improve marketers’ understanding of impulse buying behaviors by evaluating the connection between materialism and negative affect, which will allow them to plan effective marketing strategies to increase future impulse buying and profits.

## 1 Introduction

In the contemporary century, impulse buying has become commonplace in both traditional and digital commerce (Yang et al., 2021). Along with findings from previous research articles, the actual growth of online shopping in Vietnam in recent years should be emphasized. The value of Vietnam's e-commerce market reached around 12 billion US dollars in 2020, and the current digital population and increasing Internet penetration provide favorable conditions for e-commerce enterprises to expand further (Statista, 2021). The tendency toward online consumption is growing among the younger generation, who are more susceptible to impulse buying for a variety of reasons. The primary drivers of impulse buying consist of characteristics (e.g., physical feelings, impulse-buying desires), reasons (e.g., practicality, emotionality), consumer assets (e.g., time, wealth), and sales promotions (Iyer et al., 2020). Initially, as Rook and Hoch, 1985 claimed, it was people, not products, who desired consumption. They also stated that impulsive individuals were more willing to make impulse purchases. Moreover, there has always been a long history of people comparing themselves to others, which later evolved into social comparative theory. This social behavior has facilitated people living together as cohesive groups, learning from others, and reaching their full potentials. Festinger's (1954) social comparison theory and Xia et al.’s (2004) study of price equity examined the causes of these behaviors. Individualized social comparison is common in human society (Coyne et al., 2017) and influences individual conduct. This dictates what people do and appear compelled to do. Therefore, it allows us to understand social group interactions (Want and Saiphoo, 2017). According to Le (2020), the connection between social comparison and materialism can lead to impulse buying behaviors. Tokgoz (2020) showed that materialistic values had significant and beneficial effects on status, impulsiveness, and compulsive consumption.

Studies focused on the effect of social comparison on materialism (Islam et al., 2018) and negative affect (Charoensukmongkol, 2018; Liu et al., 2019a, Liu et al., 2019b; Moyal et al., 2020). For example, Liu et al., 2019a, Liu et al., 2019b indicated that upward social comparison on social media can lead to a series of negative outcomes such as malicious envy (Charoensukmongkol, 2018; Moyal et al., 2020), depressive symptoms (Li, 2019), and social anxiety (Jiang et al., 2020). The explanation is that people who experience negative emotions are more likely to engage in impulse shopping (Liu et al., 2019a, Liu et al., 2019b). Feng et al., 2021. illustrated that in hospitality, a high similarity between a reviewer and readers increases the latter's social comparison tendencies, which induced malicious envy when the writer was considered undeserving of luxury hotel consumption. Islam et al. (2018) found that social comparison was a key determinant of materialistic values; however, they only focused on downward social comparisons among individuals. Several studies examined intrinsic factors that affect impulse buying such as materialism (Oztürk and Nart, 2016; Barakat, 2019; Mukhtar et al., 2021), shopping enjoyment tendencies (Badgaiyan and Verma, 2014; Barakat, 2019; Febrilia and Warokka, 2021), marketing-driven factors (Mehta and Chugan, 2013), price and product-related factors (Jones et al., 2003; Hasanpoor et al., 2019), and internal and external triggers (Chavosh et al., 2011; Iyer et al., 2020; Kimiagari and Malafe, 2021). Mukhtar et al. (2021) found that materialism had a significantly strong and positive influence on impulse buying. However, their research included women respondents only and was thus not entirely representative because it was one-sided. Furthermore, Oztürk and Nart (2016) conducted a study on university students and found that materialistic traits substantially and favorably impacted impulse buying. In terms of confidence, which was considered a moderating variable, Mukhtar et al. (2021) stated that confidence moderated the connection between materialism and impulse buying through depression. It was explained that more confident customers were less afraid to make purchasing decisions for themselves and were less influenced by contextual factors, which led to less depression and impulse buying.

This study, based on the previous theoretical background, and using solvable problems and actual situations, aims to determine the interplay between social comparisons, materialism, negative affectivity, and impulse buying as variables and uses confidence as the moderating variable in the context of online shopping in Vietnam. Specifically, this study's goal is to explore the impact of social comparison on materialism and the effect materialism has on impulse buying and whether it is positive or negative. It also examines the moderating effect of confidence on the relationship between social comparison and materialism and the link between social comparison and negative affect.

This study contributes in a variety of ways. First, a framework model was developed to empirically examine the relationships between materialism, social comparisons, impulse buying and negative affect and how the moderating variable confidence works. In particular, this study will explain why customers' social comparisons will positively or negatively affect their levels of materialism or have no affect at all. Additionally, it contributes to determining the effect of consumers' materialism on negative affect and impulse buying, and which additional factors affect impulse buying apart from social comparisons. It extends Brown's (2016) findings that highly materialistic Vietnamese customers are willing to spend more money than consumers who are less materialistic. Moreover, this study also contributes to the theoretical framework of how impulse buying is affected by consumers' negative affect and whether negative affect was the direct cause. It expands literature on negative affect by evaluating it as the mediating factor, which is a different perspective to that of Liu et al., 2019a, Liu et al., 2019b. This study also expands the literature on consumer behavior by exploring the factors that affect impulse buying behaviors in the south of Vietnam, which other authors have not yet discovered. Furthermore, the results of this study will help improve marketers' understanding of consumer impulse buying behaviors by understanding the relationship between materialism and negative affect, and to formulate effective marketing strategies to boost impulse buying to their benefit. Apart from these contributions, this study also has some limitations. The model's results may vary because of differences between regions; hence, the study's results might not be valid elsewhere. Additionally, the study's findings may differ depending on the target participants.

This paper is presented as follows: The theoretical foundation and a review of past studies are covered next. Thereafter, the data and techniques are presented and is followed by a summary of the empirical findings. Subsequently, the findings are discussed and the final section presents the managerial implications, research limitations, and conclusion.

## 2 Literature review and hypotheses

### 2.1 Social comparison theory

The term “social comparison” was originally coined by Festinger (1954), who was the first to develop a systematic framework. However, the basic principle has been around since social philosophy and scientists have been around. The process of thinking about some factor concerning another or several other people, in reference to the self, is defined as social comparison (Meier and Schäfer, 2018). Social comparison theory is based on the concept that people have internal needs to evaluate themselves by comparing their perspectives and abilities to those of others (Usmani and Ejaz, 2020). Individual conduct is influenced by social comparison, which dictates what a person can do and considers necessary to do. Therefore, it is easy to define a social group's interpersonal affects (Want and Saiphoo, 2017). Additionally, Liu et al. (2017) explained that individuals engage in social comparison because they have access to information on other people. Comparing yourself and your relative position to others has an impact on the other person’ self-concept, level of motivation, and sense of well-being, among other factors (Suls and Wheeler, 2000). There are two types of social comparisons: upward and downward. Upward comparisons are usually connected to negative self-affects (e.g., feeling inferior), whereas downward comparisons are usually related to positive self-affects, such as feeling morally superior (Buunk and Gibbons, 2007). In theory, upward social comparisons lead to people having many negative thoughts about themselves because it reinforces the belief that others are better off than them (Schmuck et al., 2019). This form of social comparison puts one's own self in jeopardy and elicits unpleasant feelings (Jankowski and Takahashi, 2014). In contrast, downward comparisons are most frequently performed for the sake of self-improvement (Luo et al., 2018). This form of comparison generates positive feelings (Jankowski and Takahashi, 2014).

### 2.2 Materialism

Materialism is defined as the value a person places on acquiring and having material possessions based on desires or needs, and the type of behavior they engage in to achieve the desired results (Richins and Dawson, 1992). Materialism was described by Belk (1984) as “the significance a consumer gives to worldly belongings.” Richins and Dawson (1992) adopted a different approach to materialism. They found that materialistic customers evaluated their achievements based on the merits of their belongings. Consumers who valued materialism, had lifestyles which centered on acquiring goods (Rokeach, 1973), and they defined their goals and objectives in life in accordance with their achievements (Daun, 1983). Additionally, the interplay between materialism, social stratification, post-materialism, and consumption were investigated (Wang, 2016). Pinto et al. (2017) pointed out that materialism developed during adolescence and was impacted by extrinsic variables such as sex, age, socioeconomic status, self-esteem, friends, and classmates. In the development of globalization, one of the most important rising movements in humanities and social sciences is new materialism. However, it is one of the least understood (Gamble et al., 2019).

### 2.3 Negative affect

Fear, anger, sadness, guilt, and disgust are examples of negative affect, which is an underlying feature of a wide range of emotional states (Wolniewicz et al., 2018). According to Bleil et al. (2008), negative affect has physiological correlations that are similar to sadness and anxiety, such as poor heart-rate variability. Thus, in this study, the term “negative affect” is defined as words indicating bad moods such as depression and stress. Liu et al., 2019a, Liu et al., 2019b examined the link between upward social comparison on social networking sites and impulse buying, as well as the mediating roles of negative affect and ruminating. This study emphasizes the interplay between social comparison, materialism, negative affect, impulse buying, as well as confidence, as the moderating variable between these relationships. First, it is considered whether social comparison has any impact on materialism and the negative influence of fierce competition in today's market economy. Examining these connections will help find a deeper connection between materialism and its negative influence on consumers' current impulse buying. O'Brien (2018) conducted a study in US and pointed out that food, clothing, personal care products, and shoes account for $5,400 in annual impulse expenditures. Furthermore, Verplanken et al. (2005) claimed that the objective of an impulse purchase was to make oneself feel much better. This may potentially boost chronic negative sensations. Negative affect has been demonstrated to contribute to both the cognitive and affective components of impulse buying.

### 2.4 Impulse buying

Consumers engage in impulsive consumption when they acquire something unintentionally, without thinking, and without planning (Serfas et al., 2014). Furthermore, unplanned, and unexpected buying, also known as impulse buying, are frequently accompanied by cognitive and emotional reactions (Rook, 1987). According to Beatty and Ferrell (1998), consumers took more care when purchasing highly expensive items, and were more likely to be impulsive when acquiring cheaper products. In general, consumers' impulse buying tendencies were instinctive, and indicated a general inclination for impulse buying. Previously, impulsive customers valued online reviews for their hedonic values, whereas now impulsive customers value online reviews for their utility values (Zhang et al., 2018). However, impulse buying was found to be similar to rational decision making, which had long been an assumption in consumer behavioral research (Verhagen and Van Dolen, 2011). According to previous research, many factors affect impulse buying. The four components that particularly contribute to impulse buying are external cues, internal stimuli, situational and product-related factors, and demographic and socio-cultural factors (Muruganantham and Bhakat, 2013). External stimuli are marketing indicators used by marketers to entice customers to make a purchase (Yoon and Faber, 2000), whereas internal stimuli are aspects of a person's personality (Luo et al., 2018). Furthermore, in the context of live-stream commerce, social presence also affects impulse buying (Ming et al., 2021). Mukhtar et al. (2021) also found that consumers' materialistic values predicted impulse buying and distress among consumers in Pakistan.

### 2.5 Confidence

According to Davis, a sense of faith in one's talents, traits, and judgment is characterized as confidence. Mukhtar et al. (2021) showed that risk, as well as psychological traits such as self-confidence and self-esteem, are all aspects that influence buying decisions. Consumers who lacked confidence depended more on external information obtained from others to evaluate products because their lack of confidence and risk aversions resulted in them being unable to assess matters for themselves (Khan et al., 2016). Moreover, self-confidence refers to how people feel about themselves, how they differ from others, and their talents and competencies in general. However, confidence is based on people's activities in which they demonstrate self-esteem through their actions. In addition, consumers bought on impulse when they experienced passionate afflictions, such as anxiety, mental distress, disappointment, agony, and stress (Weinstein et al., 2016). This shows that when people have high levels of product involvement or understanding of technical specifications, their emotions are activated, and product involvement becomes a significant component in encouraging impulse-buying behaviors (Mukhtar et al., 2021).

### 2.6 Research framework

**Figure 1:**
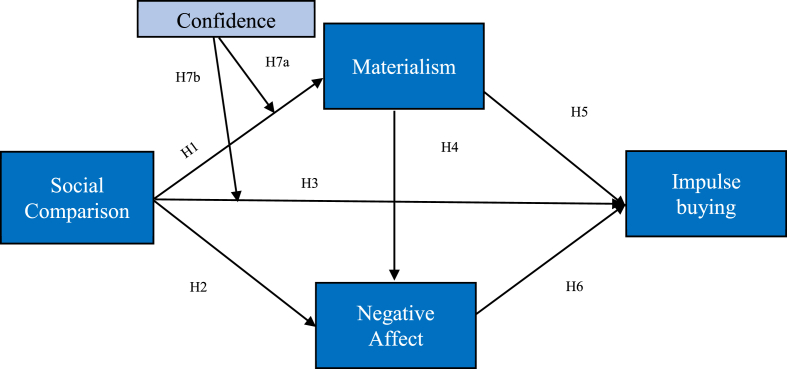
Proposed model.

The central research proposal of this study is based on a combination of the preceding findings and includes exploring the interaction of these variables, which are social comparison, materialism, and negative affect, on impulse buying and using confidence as the mediator. This model with the mediator variables and confidence is unique and will provide a deeper understanding of the relationship between materialism and negative affect on impulse buying. Liu et al., 2019a, Liu et al., 2019b examined the association between upward social comparison, materialism, and negative affect. In addition, potential factors, such as materialism, negative affect, and social comparison positively influenced impulse buying. However, this study also pointed out certain limitations other than those that are common in social research, such as a narrow research area and a small population size. The study mostly focused on the mediating effect of chronic negative emotions in the relationship between upward social comparison, on social networking sites, and impulse buying. Measuring upward social comparison on social networking sites and negative affect simultaneously may prime individuals’ emotional reactions to social comparison on social network sites, even though participants were instructed to indicate their general reactions. Additionally, other potential factors were considered as mediating factors within these relationships. Furthermore, this study examined the negative impact of social comparison, which could lead to depression (Lee et al., 2020; Pang, 2021), envy (Moyal et al., 2020; Latif et al., 2021) and the positive impact of social comparison on impulse buying (Beatty and Ferrell, 1998). Mukhtar et al. (2021) observed the moderated effect of confidence as a valuable reference. In particular, they found that confidence moderated the relationship between materialism and impulse buying through depression. However, previous studies have not studied the moderating effect of confidence between social comparison and materialism or how confidence affects the relationship between social comparison and negative affect. Therefore, this study proposes an appropriate conceptual framework (see Figure 1).

### 2.7 Hypothesis

According to Díaz and Arroyo (2017), social comparison has a significantly positive effect on materialism. In line with Gu and Hung's research (2009), social comparison, incorporating media celebrity imitation, is a fundamental driver of materialism. La Ferle and Chan (2005) also discovered that social comparison was a fundamental driver of materialistic values, and it is well recognized that materialistic tendencies affected compulsive buying among adolescents (Reeves et al., 2012). Furthermore, Zheng et al. (2018) pointed out that upward social comparisons increased both benign and malignant envy, resulting in increased materialism. Tatzel (2002) found that consumers had good attitudes toward debt and impulse shopping, and their spending requests and density of materialism were high. Belk (1995) found that materialistic consumers were fascinated by or addicted to spending, and this propensity implied buy now – think later behaviors. Furthermore, Chatterjee et al. (2019) discovered that upward social comparisons were associated with blatant materialism. Leavitt et al. (2019) gave an example of how social comparison could boost materialism. In their study, American and Brazilian women were shown items purchased by their families or friends, which led to them wanting to purchase similar items. Previous research mostly focused on adolescents; however, this study focused on adults' materialism, which can be construed as more representative of the whole population. Therefore, the following hypothesis is proposed:

H1 Social comparison has a positive impact on materialism

Social comparisons can have several harmful consequences on social media. Robinson et al. (2019) indicated that online social comparison can have negative effects on an individual's self-evaluation and level of distress. In particular, Appel et al. (2015) showed that comparing oneself to the unrealistically positive profiles of others, on social networking sites, can cause depressive symptoms. However, the study did not clarify specific social comparisons, namely, upward, or downward comparisons. Nesi and Prinstein (2015) showed that upward social comparison on social media sites was linked to depressive symptoms. Furthermore, Liu et al., 2019a, Liu et al., 2019b focused on upward social comparison and showed that negative affect played a pivotal role in evaluating upward social comparison on online communication to forecast impulse buying. Additionally, Liu et al. (2017) revealed that upward social comparison on social networking sites was a significant predictor of depressive symptoms among Chinese undergraduate students. In terms of downward comparison, pleasurable feelings of pride and amusement (Smith, 2000) or the negative emotions of pity (e.g., Wood and Vander Zee, 1997) could emerge from downward comparison. People sensed pity, dread, anxiety, empathy (assimilative emotions), scorn, or pride after downward social comparisons (contrastive emotions) (Rosenthal et al., 2019). These studies show that there are not many studies on general social comparisons, which target the adult population. Therefore, this study hypothesizes that social comparison in general can cause negative affect such as pressure or feelings of inferiority, which will influence adults in making social comparisons. Accordingly, the following hypothesis is proposed:

H2 Social comparison has a positive impact on negative affect

According to Buunk and Gibbons (2007), comparison targets suggest that individuals with similar connected traits were more likely to be compared. They showed that self-image comparison was a significant aspect of social comparison. In particular, those subjected to upward comparison were very anxious about their appearances and selves, which led to an upsurge in impulse buying. In contrast, Lucas and Koff (2017) discovered that comparing one's physical appearance to someone else's could enhance impulse buying. Additionally, impulse buying could be triggered by social comparisons, such as when customers observed their peers buying certain goods (Liu et al., 2019a, Liu et al., 2019b; Zafar et al., 2021). However, this study only looked at middle-school children rather than a clinical sample, which could be considered a limitation. Thus, the following hypothesis is proposed:

H3 Social comparison has a positive impact on impulse buying

Preliminary studies connected materialism to a slew of negative personal consequences, including unhappiness about life, poor marital satisfaction, overconsumption, and diminished well-being (Wang et al., 2017). Moreover, Roberts and Clement (2007) illustrated that negative affect from materialism included unhappiness with life in general, discontent with socialization, nervousness, excessive purchasing, low financial well-being, and so on. Furthermore, Muñiz et al. (2019) found that there was a positive relationship between materialism and depressive symptoms. However, despite the descriptive nature of their research methodology, they were unable to determine if materialism caused a greater inclination toward sadness and unhappiness, or the converse. Furthermore, studies on materialism mainly emphasized what people generally value in life or in specific life domains, such as athletics (Vansteenkiste et al., 2004b), education, and family (Vansteenkiste et al., 2004a). In addition, Dittmar et al. (2014) found that materialistic circumstances may have mitigated the negative impacts of individual materialistic values. Sirgy (1998) pointed out that the significant negative relationship between materialism and life happiness was mediated by evaluating the standards of living. Accordingly, it is reasonable to offer this hypothesis based on the above findings.

H4 Materialism has a positive impact on negative affect

According to several studies, highly materialistic consumers have distinct consumption patterns that are determined by their perceived social statuses. They have heightened senses of social status and consume status items. In other words, impulse buying is influenced by materialism, particularly among younger consumers, who have larger discrepancies between their actual and ideal selves and are more likely to buy to reinforce and affirm their self-concepts (Moran, 2015). According to Türk and Ercis (2017), impulse buying and its connection to materialism is a psychological phenomenon among young adults in Turkey. In particular, materialists have such a high proclivity for consumer buying because an increase in materialism leads to a corresponding increase in impulse buying. Similar to that study, Sen and Nayak (2019) discovered that Indian youngsters were materialistic and consequently engaged in impulse shopping. They claimed that materialism increased people's needs for material belongings and lured them to buy in excess, with a significant percentage being impulsive. However, the study was conducted in the Eastern market, which may differ from the Asian market. In addition, (Yi and Tai, 2020) indicated that those who envision unfavorable consequences purchasing an item would be less likely to engage in impulsive purchases in the future and could become less materialistic. In contrast those who imagine favorable outcomes for their purchase decisions would be more likely to buy impulsively again and become more materialistic. Thus, the hypothesis is as follows:

H5 Materialism has a positive impact on impulse buying

### 2.8 Negative affect

The link between negative mood and impulse buying has been validated by a large number of studies (Liu et al., 2019a, Liu et al., 2019b). Verplanken et al. (2005) claimed that negative affect contributed to both the cognitive and affective components of impulse buying. Depression and dissatisfaction were also found to be positively connected to impulse buying by Sneath, Lacey and Kennett-Hensel (2009). In terms of snack consumption, Romagnoli et al. (2021) discovered that negative affect was the main driver of occasional snack impulse buying. However, that study's main limitation was that it was conducted in the field of direct consumption, such as buying at the market or convenience stores, which might not represent other industries. The negative affect scale was used to assess participants' moods; however, performing this procedure might have influenced their subsequent conduct. Furthermore, Broadway et al. (2020) also showed that negative affect situations might trigger impulse buying, as evidenced by consumers who claimed to have used impulse shopping to alleviate their depressed mood states. However, this study highlighted their limitations as demographic gaps. In addition, Liu et al., 2019a, Liu et al., 2019b supported the earlier finding that negative affect, such as guilt and regret were linked to impulse buying. Many researchers discovered that negative affect predicted impulse buying as a way of dealing with negative emotions (Silvera et al., 2008). In other words, impulse buying results in people escaping from negative feelings (Liu et al., 2019a, Liu et al., 2019b). Hence, the following hypothesis is proposed:

H6 Negative affect has a positive impact on impulse buying

### 2.9 The moderating effect of confidence

Chuang et al. (2013), in a psychology study, found that confidence was an important component of any human mental state and was a significant feature in the customer purchasing decision-making process. Consequently, it is frequently utilized to analyze consumer behavior. Confidence is one of the most essential identity attributes, which determines how individuals react to circumstances. These reactions and activities then determine their mindsets (Benabou and Tirole, 2002). Thus, if shoppers had more confidence, they would be less afraid to make purchasing decisions for themselves and would be less influenced by outside information, which would reduce despair and impulse buying (Mukhtar et al., 2021). However, the more information a customer seemed to have, the more difficult it appeared for them to make decisions (Outreville and Desrochers, 2014). Thus, the question remains whether confidence influences the relationship between social comparison and impulse buying. In contrast, consumers who lack confidence depend more on external information obtained from others to assess factors because they are unable to rate objects owing to their lack of confidence and willingness to take risks. Under materialism, the question remains whether confidence moderates the relationship between materialism and social comparison. Therefore, these hypotheses are proposed:

H7a Confidence moderates the relationship between materialism and social comparison

H7b Confidence moderates the relationship between impulse buying and social comparison

## 3 Methodology

### 3.1 Methodology used

#### 3.1.1 Participants and procedure

This study chose a youthful group of people aged 18–31 years as participants and divided them into two main groups: students from Banking University and office workers who engage in unplanned impulse buying. According to Nielsen's Vietnam study (2018), 60 percent of online buyers are women and 55 percent are between the ages of 25 and 29, which validates our choice of target respondents. Aside from associating this age group with social networking sites, shopping for products online has become extremely prevalent (Temkin, 2009). Hence, during June to August 2021, 400 potential respondents were contacted to complete the survey, which was written in Vietnamese, and asked for demographic information (such as name, gender, education, and income). Hair et al. (1998) recommended that the sample size should be at least five times larger than the number of variables in the factor analysis. Consequently, 249 valid responses were obtained. All respondents were residents of Ho Chi Minh city. The data was collected during the course of one month, starting from August 1, 2021.

Pilot and actual testing were conducted in two stages. The questionnaire was pilot tested with a sample of ten respondents over the course of two days prior to the actual testing. The primary goal of the pilot test was to detect troublesome questions. This is a chance for the questionnaire designer to learn whether there is any uncertainty about any of the items as well as whether participants have any recommendations on how the items can be improved (Tsang et al., 2017). Consequently, the author assessed the questionnaire, in the pilot test, based on a number of factors, including its relevance, conciseness, and practicality, as well as language and item sequencing (Buschle et al., 2021). Fortunately, there were no concerns about the clarity of the items or the questionnaire's acceptability. In terms of the official test, this study collected online responses from Vietnamese residents via social networking sites such as Zalo, Facebook, and Gmail. Online surveys were conducted because they are cost-effective and quick (Nayak and Narayan, 2019). Furthermore, because of the significant risk of transmitting COVID-19, this technique permitted the author to obey the government's admonition to stay at home. In terms of research area, this study was conducted in Ho Chi Minh city and it was chosen because in 2021 the city had 35.4 million e-commerce consumers in Vietnam, who made approximately 6.6 million worth of purchases online.

#### 3.1.2 Questionnaire design

This study used existing measures and a 5-point Likert scale was used to measure these items. For demographic variables, gender, age, and monthly living expenses have all been linked to impulse buying (Coley and Burgess, 2003; Vohs and Faber, 2007). Consequently, these factors were used as control variables. To measure social comparison, modified items from the Likert scale developed by Liu et al., 2019a, Liu et al., 2019b and Wang et al. (2017) were used. Moreover, ten negative impact items were distributed randomly based on Watson et al. (1988). The impulse buying scale was used to assess impulse buying as developed by Verplanken and Herabadi (2001), Vazquez et al. (2020), and Zhang et al. (2018). Materialism was assessed using the 9-item scale developed by Mukhtar et al. (2021), Pradhan et al. (2018), and Le (2020). Moreover, confidence was observed using five items based on Dash et al. (1976). Some measurement items were the author's own creations based on the current situation in Vietnam and the author's own experience of Vietnamese people's ethnicity, culture, and characteristics. These measurements included: “I habitually compare myself to others,” “My regular topic of conversation is how I compare to others,” “I often think that other people are happier,” and “I am ashamed that I am less successful than my friends”. Table 1 will show all detailed measurement items.

**Table 1:** Constructs and measurement items.

Construct	Items	Measures	Supporting References
Social comparison	SC1	I always compare the way I perform tasks to the way others perform tasks.	Liu et al., 2019a, Liu et al., 2019b
	SC2	In social situations, I am prone to comparing how I look to those who are more attractive than me.	
	SC3	I frequently compare my achievements in life to others.	
	SC4	I attempt to discover other people's views on things I want to learn more about.	
	SC5	I enjoy discussing common interests and experiences with others.	
	SC6	I am always fascinated by what others might do in a similar scenario.	
	SC7	I often compare myself to people close to me (boyfriends or girlfriends, family members, etc.)	Wang et al. (2017)
	SC8	I am obsessed with comparing myself to others.	Author proposed
	SC9	My regular topic of conversation is how I compare to others.	
	SC10	I habitually compare myself to others.	
	SC11	I often think that other people are happier.	
Impulse buying	IB1	I am extremely excited when I see something that I want to buy.	Verplanken and Herabadi (2001)
	IB2	When I buy something, it is usually spontaneous.	
	IB3	I often buy things online without thinking.	
	IB4	If I see something new, I want to buy it.	
	IB5	My purchases are always unplanned.	
	IB6	I occasionally feel bad about purchasing something.	Vazquez et al. (2020)
	IB7	I occasionally purchase items that I do not need because I enjoy buying them.	Olsen et al. (2016)
	IB8	I am defined by “If I see it, I buy it.”	Zhang et al. (2018)
Materialism	MAT1	I admire people who have luxury homes, automobiles, and clothing.	Mukhtar et al. (2021)
	MAT2	I enjoy owning items that make others notice me.	Le (2020)
	MAT3	I often worry about not being able to afford everything I want to buy.	
	MAT4	My possessions reveal a great deal about how well I am doing in life.	
	MAT5	I adore splurging on items that are not useful.	Pradhan et al. (2018)
	MAT6	I value material possessions less than most other people I know.	
	MAT7	I think my life would be better if I had some of the things, I do not have.	
	MAT8	I would not be much happier if I had nicer possessions.	
	MAT9	I have all I require to live a happy life.	
Negative affect	NE1	I become irritated when I see other people's accomplishments.	Meier and Schäfer (2018)
	NE2	It does not seem fair that some people appear to be having more fun than me.	Tandoc et al. (2015)
	NE3	It makes me nervous to realize that my peers are better than me.	Charoensukmongkol (2018)
	NE4	It is depressing to realize that my peers are more successful than me.	
	NE5	It hurts to realize that someone has a better life than me.	
	NE6	I do not like it when my peers are more attractive than me.	
	NE7	I am ashamed that I am less successful than my friends.	Author proposed
	NE8	I am jealous that my peers are more successful than me.	
	NE9	I harbor a grudge (resentment, malice) that my peers are more successful than me.	Moyal et al. (2020)
	NE10	I definitely want everything that someone else has.	
Confidence	CD1	In general, I am confident of my talents.	Dash et al. (1976)
	CD1	In general, I am confident in what I am currently doing.	
	CD1	I don't regret anything that has happened to me.	
	CD1	I am confident that I am better than others.	Author proposed
	CD1	In general, I am confident about my decisions.	

## 4 Results and findings

### 4.1 Demographic statistics

The questionnaire was distributed to 260 respondents using a Google form and 249 valid responses were received. The 11 invalid responses were because the respondents incorrectly answered the reverse-scale questions. The genders were equally divided; however, most respondents were younger than 30 years. The random sample showed that men and women were equally impulsive, and those aged 31–40 years were most likely to make impulse purchases. The demographic statistics are shown in Table 2.

**Table 2:** Response rate of groups.

Category	Number of respondents	Percentage
**Gender**		
Male	115	46%
Female	134	44%
**Age**		
Less than 20	17	7%
20–30	55	22%
31–40	65	26%
41–50	63	25%
51–60	30	12%
Above 60	19	8%
**Occupation**		
Student	87	35%
Financial/insurance	67	27%
Education/culture	53	21%
Government	13	5%
Media	19	8%
Others	10	4%
**Education**		
Senior High Diploma or Below	10	4%
Associate Bachelor Degree	93	37%
Bachelor Degree	87	35%
Master Degree	49	20%
PhD Degree	10	4%

### 4.2 Ethical approval

The author received ethical approval from the Banking University Research Ethical Board, and the study complied with ethical standards although a number was not allocated to the approval. Respondents were informed both verbally and in writing about the purpose of the research, and their consent was obtained before filling out the questionnaire. Respondents were aware that their participation in the research was voluntary. They were also assured that their responses would be kept confidential.

### 4.3 Confirmatory factor analysis (CFA)

Bagozzi and Foxall (1996) showed that confirmatory factor analysis (CFA) can be used to evaluate reliability and validity. The CFA's goodness-of-fit was used to further examine the construct's convergent validity. The following indices were used in the CFA: Chi-square/df (cmin/df) = 1.941, goodness of fit index (GFI) = 0.822, adjusted goodness of fit index (AGFI) = 0.821, comparative fit index (CFI) = 0.929, root mean squared error of approximation (RMSEM) = 0.062, and Tucker Lewis Index (TLI) = 0.923. These indicator results show that a GFI greater than 0.8 and less than 0.9 is considered acceptable according to two studies by Baumgartner and Homburg (1996) and Doll et al. (1994) because it significantly depends on the scale's measure, number of observations, and sample size. Consequently, all variables in this study were within the acceptable range (see Table 3).

**Table 3:** Confirmatory factor analysis.

Measure	Threshold	Results	Source
Chi-square/df (cmin/df)	≤2 good; ≤ 3 sometimes permissible	1.941	Hair et al. (2010)
Goodness of fit index (GFI)	≥0.9: acceptable; ≥ 0.8: marginal	0.822	Hair et al. (2010)
Adjusted goodness of fit index (AGFI)	≥0.8	0.821	Hair et al. (2010)
Comparative fit index (CFI)	≥0.95 great; ≥0.90 traditional; ≥0.80 sometimes permissible	0.929	Hair et al. (2010)
Root mean squared error of approximation (RMSEM)	≤0.05 good; ≤0.08 moderate	0.062	Hair et al. (2010)
Tucker Lewis Index (TLI)	≥0.90	0.923	Hair et al. (2010)

**Construct validity**: According to Hair et al. (2009), average variance extracted (AVE) scores should be around 0.5 and should explain 50% or more of the variance. The reliability of all the variables, including social comparison, materialism, negative affect, and impulse buying, ranged from 0.912 to 0.944 (see Table 3). Therefore, the reliability of these findings is satisfactory. The factor loading of most items exceeded 0.5 (Hair et al., 2009). However, some items were rejected for being less than 0.5 including three social comparisons (SC), two materialisms, one negative affect, and one impulse buying. Moreover, AVE for each construct was greater than 0.5, and the construct reliability (CR) of all the latent variables was higher than 0.7. All indicators had significant loading into the respective latent constructs, with values between 0.588 and 0.707. Therefore, the results were acceptable and could explain over 50% of the variance (see Table 4).

**Table 4:** Confirmatory factor analysis (CFA) fitting Indices.

	Estimate	Cronbach	CR	AVE
Social comparision		0.922	0.927	0.600
SC1	0.696			
SC2	0.698			
SC3	0.654			
SC4	0.814			
SC5	0.843			
SC6	0.789			
SC7	0.840			
SC8	0.840			
**Materialism**		**0.912**	**0.907**	**0.588**
MAT1	0.709			
MAT2	0.604			
MAT3	0.669			
MAT4	0.709			
MAT5	0.865			
MAT6	0.883			
MAT7	0.881			
**Negative affect**		**0.923**	**0.935**	**0.644**
NE1	0.714			
NE2	0.775			
NE3	0.820			
NE4	0.778			
NE5	0.835			
NE6	0.835			
NE7	0.815			
NE8	0.840			
NE9	0.646			
**Impulse Buying**		**0.944**	**0.943**	**0.707**
IB1	0.812			
IB2	0.871			
IB3	0.908			
IB4	0.926			
IB5	0.833			
IB6	0.760			
IB7	0.761			

According to Hair et al. (2009), discriminant validity ensures that a concept measure is statistically distinctive and accurately depicts phenomena that other measures in a structural equation model miss. This was tested by comparing if the square root of AVE, in a latent construct, was higher than all the construct correlations. The results showed that the square of AVE values, for all the variables, social comparison, materialism, and negative affect were higher than the inter-construct correlations (see Table 5). Table 5 shows that the outer loading values of all indicators were higher than the values of all cross-loadings on the other constructs. Thus, the outcomes were considered appropriate.

**Table 5:** Discriminant validity.

	SC	MAT	NE	IB
SC				
MAT	0.60			
**NE**	0.151	**0.588**		
**IB**	0.180	0.501	**0.644**	
**IB6**	0.123	0.034	0.134	**0.707**

**Figure 2:**
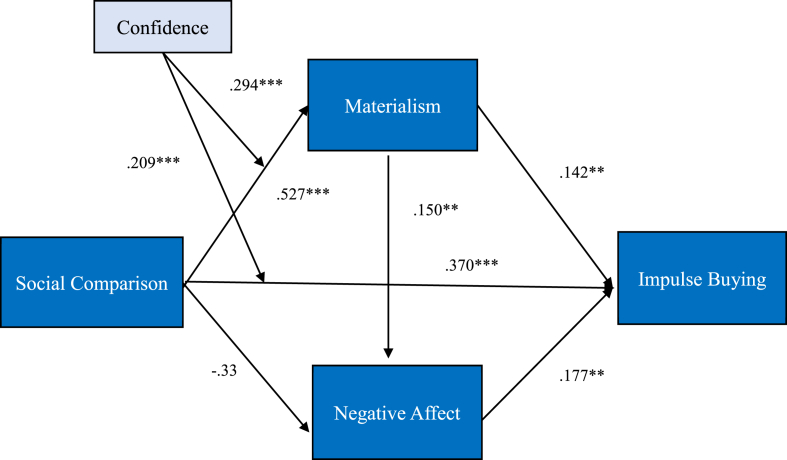
The result of the model assessment (∗∗∗p < .001).

The data analysis indicates that social comparison has a significant effect on materialism (β = 0.527, p < .001). The first hypothesis also has a t-value of 6,545, implying that people who frequently make social comparisons are also more materialistic. Thus, H1 is supported. However, social comparison does not have a significantly positive effect on negative affect (β = -0.33, t-value = -4.801), and H2 is unsupported in this study. This finding contradicts other research that if a person usually participates in social comparisons, it will not lead to negative affect, such as envy or pressure. In addition, social comparison (β = 0.370, p < .001) has a significantly positive impact on impulse buying. Highly impulsive buyers are directly affected by social comparisons. Thus, H3 is supported. Moreover, materialism is significantly positively related to negative affect (β = 0.150, t-value = 2.736) and impulse buying (β = 0.142, t-value = 2.611 p < .01). Therefore, H4 and H5 is also supported. Finally, impulse buying is positively predicted by negative affect (β = 0.177, t-value = 3.130, p < .01). Thus, H6 is also supported. Figure 2 shows the results of the model assessment and Table 6 represents the results briefing.

**Table 6:** Results of hypothesis test.

Hypotheses	Path	Standardized path coefficient	t-value	Result
H_1_	Social comparison → Materialism	0.527∗∗∗	6.545	Support
H_2_	Social comparison → Negative Affect	-0.33	-4.801	Unsupported
H_3_	Social comparison → Impulse buying	0.370∗∗∗	5.401	Support
H_4_	Materialism → Negative Affect	0.150∗∗	2.736	Support
H_5_	Materialism → Impulse buying	0.142∗∗	2.611	Support
H_6_	Negative Affect → Impulse buying	0.177∗∗	3.130	Support

Note. N = 249, ∗p < .014; ∗∗p < .01; ∗∗∗p < .001

### 4.4 Moderation results

**Figure 3:**
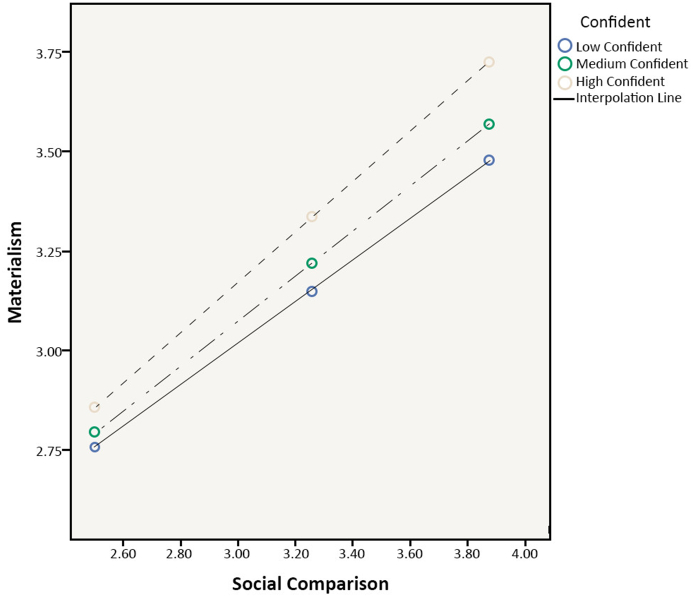
Plotted interaction of social comparison and confident on materialism.

**Figure 4:**
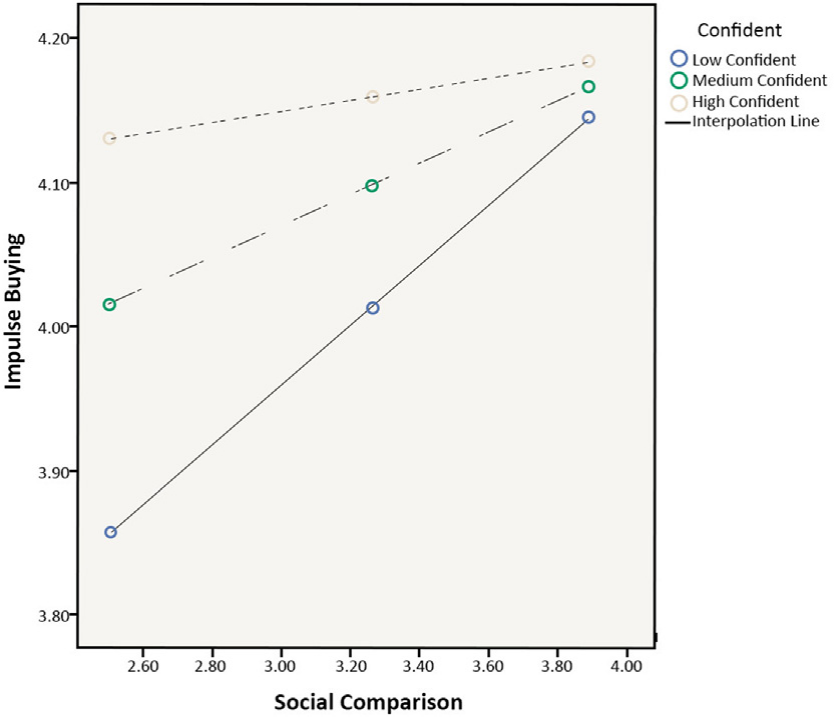
Plotted interaction of social comparison and confident on impulse buying.

The author investigated whether the impact of social comparison on materialism could be examined through confidence (moderator) and if the results support the assumption of the hypothesized moderated model (H7a was supported). Specifically, a test was first conducted to determine whether the interaction between social comparison and confidence had a significant effect on materialism. A significant interaction effect was found, β = 0.294, p < 0.001. Social comparison and confidence also had a significant interaction effect on impulse buying (β = 0.209, p < 0.001; H7b was supported). In particular, social comparison interacted with confidence to the extent that those with lower levels of confidence have materialistic and impulse buying tendencies that are very similar across low and high levels of social comparison. When making more comparisons, those with higher levels of confidence indicated higher levels of materialism and impulse buying. Similarly, regardless of materialism levels, individuals who reported lower levels of confidence had reduced impulse-buying tendencies, whereas those with greater levels of confidence reported significant impulse buying due to strong materialism. The results can be interpreted that if shoppers were more confident, they would be less afraid to make purchasing decisions for themselves, which could lead to a rise in materialism, such as strong desires to own more things, and splurging on useless stuff. This would directly impact their impulse and spontaneous purchases. The results are shown in Figures 3 and 4.

## 5 Discussion

A total of 400 surveys were sent to young individuals via the Internet. Those who had done online shopping before were asked to participate in the study. However, only 249 surveys were valid of the 400 surveys and used in the analysis. In addition, this study relied on previously-published measurements. These items were assessed using a 5-point Likert scale. Demographic characteristics such as gender, age, and monthly living expenditures were previously connected to impulse buying (Coley and Burgess, 2003; Vohs and Faber, 2007). The other main measurement constructs were based on various authors such as Watson et al. (1988), Verplanken and Herabadi (2001), Vazquez et al. (2020), and Zhang et al. (2018). In addition, some of the measures were the author's own recommendations and based on current contextual factors in Vietnam, and the author's personal opinions of factors that would demonstrate the ethnicity, culture, and features of Vietnamese consumers.

The antecedents and implications of impulse buying and social comparisons have been studied in various fields. However, few studies have examined how materialism affects impulse buying or how it is influenced by social comparison. As a result, the goal of this study was to determine the link between social comparison, materialism, impulse buying, negative affect, and confidence as a moderating factor in the model by examining eight hypotheses. The results indicate that social comparison has a positive impact on materialism, leading individuals to purchase impulsively, which is consistent with the findings of Zheng and Peng (2018). They found that after making upward social comparisons, consumers placed higher values on material things and that people were more inclined to spend money on publicly visible products compared to those making downward or no social comparisons.

The result of the positive relationship between social comparison and materialism indicates that when people compare themselves socially to those who are better than them, they will admire the better lifestyles that create strong desires for them to buy similar luxury items. In contrast, if they compare themselves to people who are inferior to them, they are likely to feel confident and proud of themselves; thus, it is easier for them to make unplanned purchasing decisions. However, this study states that there is not a significant relationship between social comparison and negative affect. In contrast, Tandoc et al. (2015) found that upward social comparison, on social networking sites, can lead to a series of negative effects. For example, people's self-perceptions and evaluations are weakened when they are exposed to images of beautiful individuals (Fardouly et al., 2015). In particular, young adults are more inclined to compare themselves to others on social media and may be more negatively affected (Shaw et al., 2013).

However, this study, in the Vietnamese context, did not confirm that people who make upward social comparisons do not suffer any negative affect such as envy or pressure. A possible explanation for finding that social comparison is not linked to negative affect is that according to Wheeler's (1966), people do not consider upward comparisons a danger to their self-esteem, but rather as an opportunity to improve themselves. Moreover, when participants compared themselves to others, especially in upward comparisons, they were normally more joyful, experienced more schadenfreude, and felt superior, relieved, and better. They were also less envious, unhappy, and felt more worthy. Their desires to be like another person were diminished (Rosenthal et al., 2019). Another explanation in the Vietnamese context is that when people often compare themselves to others, they often feel anxious, sad, judged, and jealous and their personalities are actively being restricted. This is probably the main reason this study's results do not support Hypothesis H2.

Moreover, this study also finds that there is a significant relationship between social comparison and impulse buying. This contributes to understanding Vietnamese consumer behaviors. If people who usually buy impulsively also usually compare themselves to other people they interact with, they might highly desire the things that these other people own. This finding corroborates Liu et al., 2019a, Liu et al., 2019b research in which they illustrated that making upward comparisons on social media might lead to increased impulse buying among young individuals. In addition, impulse buying can be triggered by social comparisons, such as when customers observe their peers buying certain goods (Rook et al., 1995). Roberts and Manolis (2012) also found that impulse buying was frequently triggered by social comparisons. Furthermore, this study confirmed the impact of negative affect on impulse buying, in line with many existing studies. When people experience unpleasant emotions, they are more likely to engage in impulse shopping and see it as a method to enjoy themselves (Liu et al., 2019a, Liu et al., 2019b), and this tendency also exists in Vietnam. In Vietnam, the more people feel bad, because of pressure, stress, and so on, the more prone they are to shop impulsively to feel better. Therefore, this tendency is easy to understand in terms of basic human psychological desires, which considers that psychological behaviors cause people to splurge. This was researched, in psychology, by Park et al. (2006) and Naeem (2020).

Moreover, a recent study contributes to earlier findings that materialism increases impulse buying. For example, if people strongly desire something, they will not hesitate to buy it. This is in line with Moran (2015), who revealed that there was a strong correlation between materialism and impulse consumption among women college students aged 18 to 27. In addition, Vohra (2016) found that materialistic values have a major influence on impulse buying among young customers. Moreover, Yoon and Kim (2016) demonstrated the influence of materialistic ideals on impulse consumption by using qualitative and in-depth interviews and methods, and a sample of hypermarket customers and university undergraduate students. Moreover, the study also stated the relationship between materialism and negative affect, which has not been the focus of many previous studies. Alzubaidi et al. (2021) found that materialism significantly affected consumer intentions.

Furthermore, this study examined the unique feature of confidence in the direct and indirect links between social comparison on social media and impulse buying, as well as social comparison and materialism. Thus, these findings on the moderator variable consider new points that contribute to existing theories. In particular, people with lower levels of confidence have materialistic and impulse buying inclinations that are highly comparable across low and high levels of social comparison, indicating that social comparison interacts with confidence. Furthermore, those with higher levels of confidence expressed greater levels of materialism and impulse buying and made more comparisons. In accordance with this, individuals with lower levels of confidence participated less in impulse buying and this was not based on the degree of materialism. In contrast, those with higher levels of confidence strongly engaged in impulse buying due to strong materialism.

## 6 Conclusion

In a fiercely competitive world market, customers have increasingly more choices and power, and customer psychology changes accordingly. Impulse shopping has become increasingly popular. People buy something that is not based on their original intention. Impulse buying is influenced by many direct and indirect factors. Many previous studies have shown that social comparison, materialism, and negative affect directly influence direct shopping. Therefore, this study builds a model to determine the relationship between the following factors: social comparison, materialism, negative affect, and impulse buying, and the interplay between these factors. This study also identifies the role of confidence in moderating the relationship between social comparison and impulse buying, as well as social comparison and materialism. These results are most consistent with earlier studies that investigated such variables in the context of social comparison, such as Liu et al., 2019a, Liu et al., 2019b and Zheng et al. (2018). However, this study's results do not validate the research of Fardouly et al. (2015) and Liu et al., 2019a, Liu et al., 2019b because the results reject the relationship between social comparison and negative affect. Moreover, few studies examined whether confidence played a moderating role in the relationship between social comparison and materialism. This study illustrated that confidence moderates the relationship between impulse buying and social comparison and further pointed out that consumers could not rate items themselves, owing to their lack of confidence and willingness to take chances. Consumers with less confidence depended more on external information, obtained from others through social comparison, and made assessments accordingly.

### 6.1 Managerial implications

The major findings of this study have numerous crucial implications. First, people often tend to compare themselves to others to improve their understanding of themselves and their abilities (Festinger, 1954). Consequently, the findings of this study will assist marketing managers in identifying consumer impulse-buying decision-making processes based on social comparisons, particularly in terms of materialistic qualities that encourage consumers to purchase. Managers may utilize this information to develop new goods, modify presentation styles, and brainstorm new marketing strategies. Businesses involved in e-commerce should emphasize the status aspects of their products and services and focus on marketing creative communication messages during sales promotions or direct sales because materialism, including happiness, success, and popularity (Richins and Dawson, 1992) favorably influences impulse buying (Moran, 2015). Moreover, businesses should make genuine attempts to improve their social comparisons by encouraging influencers to promote their goods, which implies that when fans see them using something, they will buy it without hesitation. Negative affect also influences impulse buying positively. This finding provides managers with deeper insights into customers. Specifically, when customers feel depressed, anxious, or nervous, they are more likely to buy impulsively.

### 6.2 Limitations

The current study has some limitations. First, because the model's outcomes may change in different contexts, the study's conclusions have low external validity, and the study's findings may also differ with a different target demographic. In addition, the research was conducted during the height of COVID-19 in Vietnam and may therefore differ if conducted in other research times and areas. Second, this study relied on a quantitative approach, which limits the scope of our findings. Thus, scholars should focus on using qualitative and longitudinal data in future research to obtain more detailed results and a more accurate image of the target population. Qualitative research is intended to produce in-depth and subjective conclusions with a small sample size (Crick, 2021). However, if it is correctly conducted, it can yield unbiased, valid, credible, and rigorous results (Anderson, 2010). Furthermore, future studies should conduct in-depth interviews or host focus groups that target those who regularly buy impulsively to gain deeper insights. In addition, various talent elements impact impulse buying in both online and traditional channels, including promotional schemes (Cho et al., 2014), and perceived utilitarian and hedonic values (Yang et al., 2021). Thus, further research could consider examining other scenarios and potential determinants. In addition, this study did not specifically investigate the components that comprise negative affect, such as malicious envy and depression. Thus, future studies could research a certain type of negative affect to obtain more specific insights.

## 7 Ethical statement

My research does not use human or animal subjects.

## Declarations

### Author contribution statement

Van Dat Tran has done: Conceived and designed the experiments; Performed the experiments; Analyzed and interpreted the data; Contributed reagents, materials, analysis tools or data; Wrote the paper.

### Funding statement

This research did not receive any specific grant from funding agencies in the public, commercial, or not-for-profit sectors.

### Data availability statement

Data will be made available on request.

### Declaration of interest’s statement

The authors declare no conflict of interest.

### Additional information

No additional information is available for this paper.
